# Activation of TGF-β signaling induces cell death via the unfolded protein response in Fuchs endothelial corneal dystrophy

**DOI:** 10.1038/s41598-017-06924-3

**Published:** 2017-07-28

**Authors:** Naoki Okumura, Keisuke Hashimoto, Miu Kitahara, Hirokazu Okuda, Emi Ueda, Kyoko Watanabe, Makiko Nakahara, Takahiko Sato, Shigeru Kinoshita, Theofilos Tourtas, Ursula Schlötzer-Schrehardt, Friedrich Kruse, Noriko Koizumi

**Affiliations:** 1 Department of Biomedical Engineering, Faculty of Life and Medical Sciences, Doshisha University, Kyotanabe, Japan; 2 Department of Ophthalmology, Kyoto Prefectural University of Medicine, Kyoto, Japan; 3 Department of Frontier Medical Science and Technology for Ophthalmology, Kyoto Prefectural University of Medicine, Kyoto, Japan; 4 Department of Ophthalmology, University of Erlangen-Nürnberg, Erlangen, Germany

## Abstract

Fuchs endothelial corneal dystrophy (FECD) is a slowly progressive bilateral disease of corneal endothelium in which accumulation of extracellular matrix (ECM) and loss of corneal endothelial cells (CECs) are phenotypic features. The corneal endothelium maintains corneal transparency by regulating water hydration; consequently, corneal endothelial dysfunction causes serious vision loss. The only therapy for corneal haziness due to corneal endothelial diseases, including FECD, is corneal transplantation using donor corneas, and no pharmaceutical treatment is available. We provide evidence that the expression levels of transforming growth factor-β (TGF-β) isoforms and TGF-β receptors are high in the corneal endothelium of patients with FECD. A cell model based on patients with FECD shows that TGF-β signaling induced a chronic overload of ECM proteins to the endoplasmic reticulum (ER), thereby enhancing the formation of unfolded protein and triggering the intrinsic apoptotic pathway through the unfolded protein response (UPR). We propose that inhibition of TGF-β signaling may represent a novel therapeutic target that suppresses cell loss as well as the accumulation of ECM in FECD.

## Introduction

Fuchs endothelial corneal dystrophy (FECD) is a slowly progressive bilateral disease of the corneal endothelium. The corneal endothelium maintains corneal transparency by regulating water hydration; consequently, severe damage to the corneal endothelium induces corneal haziness, resulting in critical vision loss. The only therapy for corneal haziness due to corneal endothelial diseases including FECD is corneal transplantation using donor corneas. The high prevalence of FECD (i.e., 4% of the population aged over 40 years in the United States^1^) makes FECD a leading cause of corneal transplantation^2^. However, the shortage of donor corneas, the difficulty of the surgical procedure, and the incidence of graft failure in both acute and chronic phases^3, 4^ encourages the development of pharmaceutical treatments.

The corneal endothelium in FECD shows features of apoptotic cells, such as positive TdT-mediated dUTP nick end labeling (TUNEL) staining, condensation of nuclei, and decreased cell size^5^. Apoptosis has been indicated to play an important role, but the pathophysiology of FECD remains unclear. Engler and colleagues reported morphological alterations of the endoplasmic reticulum (ER) in FECD and postulated that the unfolded protein response (UPR) plays an important role in inducing apoptosis^6^. Under normal conditions, secreted and membrane proteins are folded in the lumen of ER for delivery to membranes or extracellular secretion^7, 8^. Properly folded proteins are packaged into ER exit vesicles, whereas improperly folded proteins are retained in the ER and are removed by proteasomal degradation, called ER-associated degradation (ERAD)^9^. This highly regulated system is monitored by a conserved signaling pathway, termed the UPR^10, 11^. However, severe ER stress, with its associated impairment of homeostasis, induces chronic activation of the UPR, resulting in apoptosis to remove rogue cells^10, 11^. Notably, increasing evidence now indicates that the UPR is involved in the pathogenesis of various diseases, such as Alzheimer’s disease, Parkinson’s disease, diabetes mellitus, multiple myeloma, and retinitis pigmentosa^12–17^.

The phenotypic features of FECD in the clinical setting are (1) formation of corneal guttae that are excrescences of the basement membrane (Descemet’s membrane) and (2) endothelial cell loss^18^. Corneal guttae have been recognized as an abnormal accumulation of extracellular matrix (ECM) components, such as fibronectin and type 1 collagen, secreted by pathological corneal endothelium^19^. However, the mechanisms for upregulation of secretion of these ECM proteins and the association of the ECM accumulation with endothelial cell loss in FECD remain unclear. In the context of ECM production, we recently demonstrated in a cell model established from patients with FECD^20^ that transforming growth factor-β (TGF-β) regulates excessive ECM protein production.

In the current study, the expression level of TGF-β isoforms and TGF-β receptors was examined in clinical samples of FECD patient corneal endothelium. We also examined the effect of TGF-β on the formation of unfolded protein and subsequent triggering of the UPR. Finally, we evaluated the feasibility of inhibiting TGF-β signaling as a possible therapeutic target for treatment of FECD.

## Results

### Expression levels of *TGF*-*β* isoforms and receptors in the corneal endothelium of Patients with FECD

**Figure 1:**
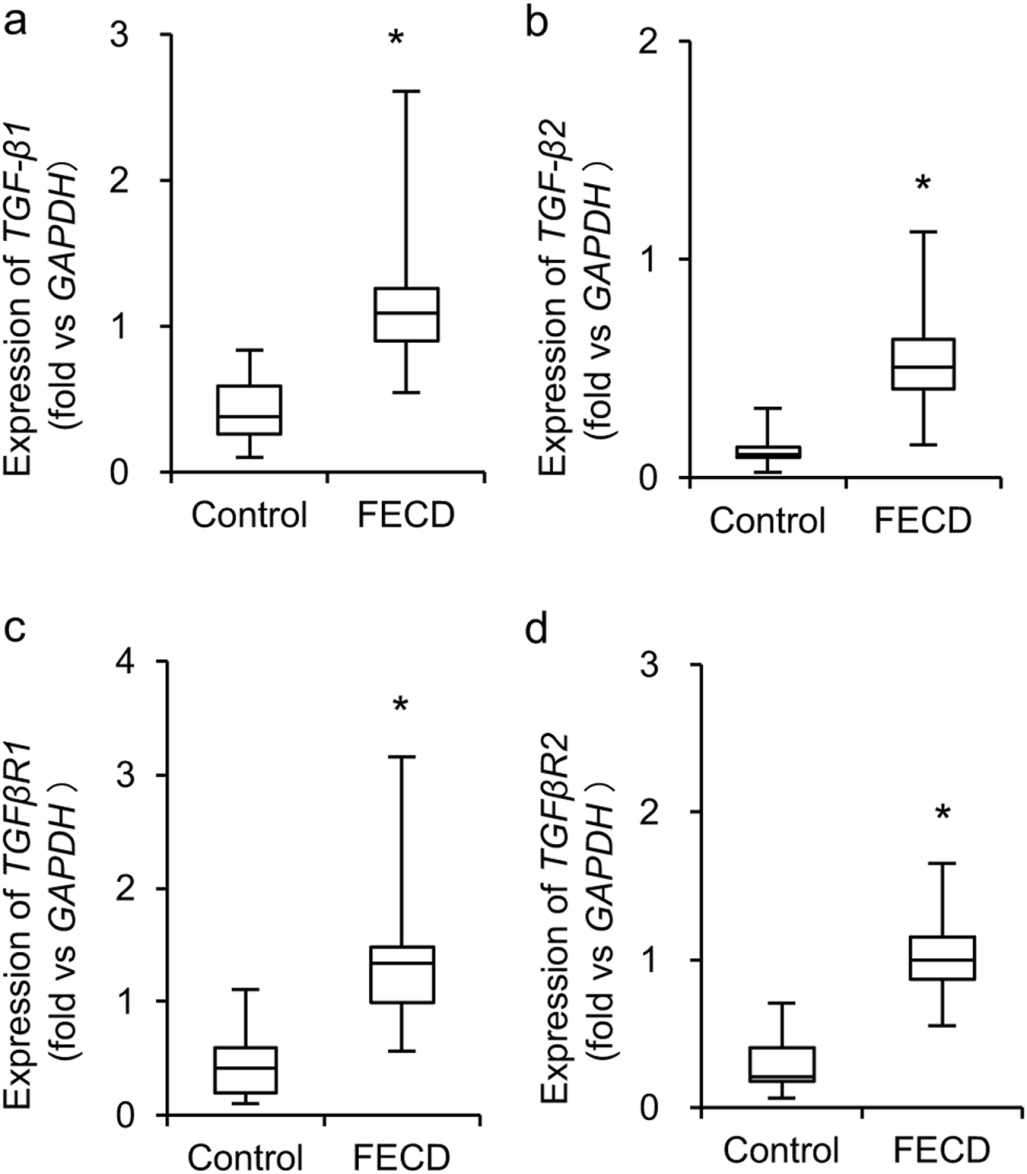
Expression of *TGF*-*β* isoforms and receptors in corneal endothelium of patients with FECD. (**a**–**d**) Descemet’s membranes with CECs were obtained from 30 patients with FECD during Descemet’s membrane endothelial keratoplasty (DMEK). Thirty human donor corneas from non-FECD subject were used as control. Expression levels of *TGF*-*β1*, *TGF*-*β2*, *TGFβR1*, and *TGFβR2* were evaluated by quantitative real-time PCR. GAPDH was used as an internal standard. The horizontal lines in the boxes indicate medians, the bottom of each box indicates the 25th percentile, and the top of each box indicates the 75th percentile. The horizontal lines outside the boxes indicate the range of mRNA level. The statistical analysis was performed by Mann–Whitney U test. *P < 0.01.

We established a cell model from patients with FECD and showed that corneal endothelial cells (CECs) have a higher responsiveness to TGF-β when compared to control CECs, which results in excessive ECM production^20^. We therefore evaluated the expression levels of *TGF*-*β* isoforms and receptors in patient samples. Quantitative real-time polymerase chain reaction (PCR) assays showed that expression levels of *TGF*-*β1* and *TGF*-*β2* were significantly higher in Patients with FECD (n = 30) than in normal control subjects (n = 30) (2.76 and 4.36 fold, respectively) (Fig. 1a,b). Likewise, expression levels of *TGF*-*βR1* and *TGF*-*βR2* were significantly higher in FECD samples than in control samples (3.03 and 3.79 fold, respectively) (Fig. 1c,d).

### Effect of TGF-β on ECM protein expression and unfolded protein formation

**Figure 2:**
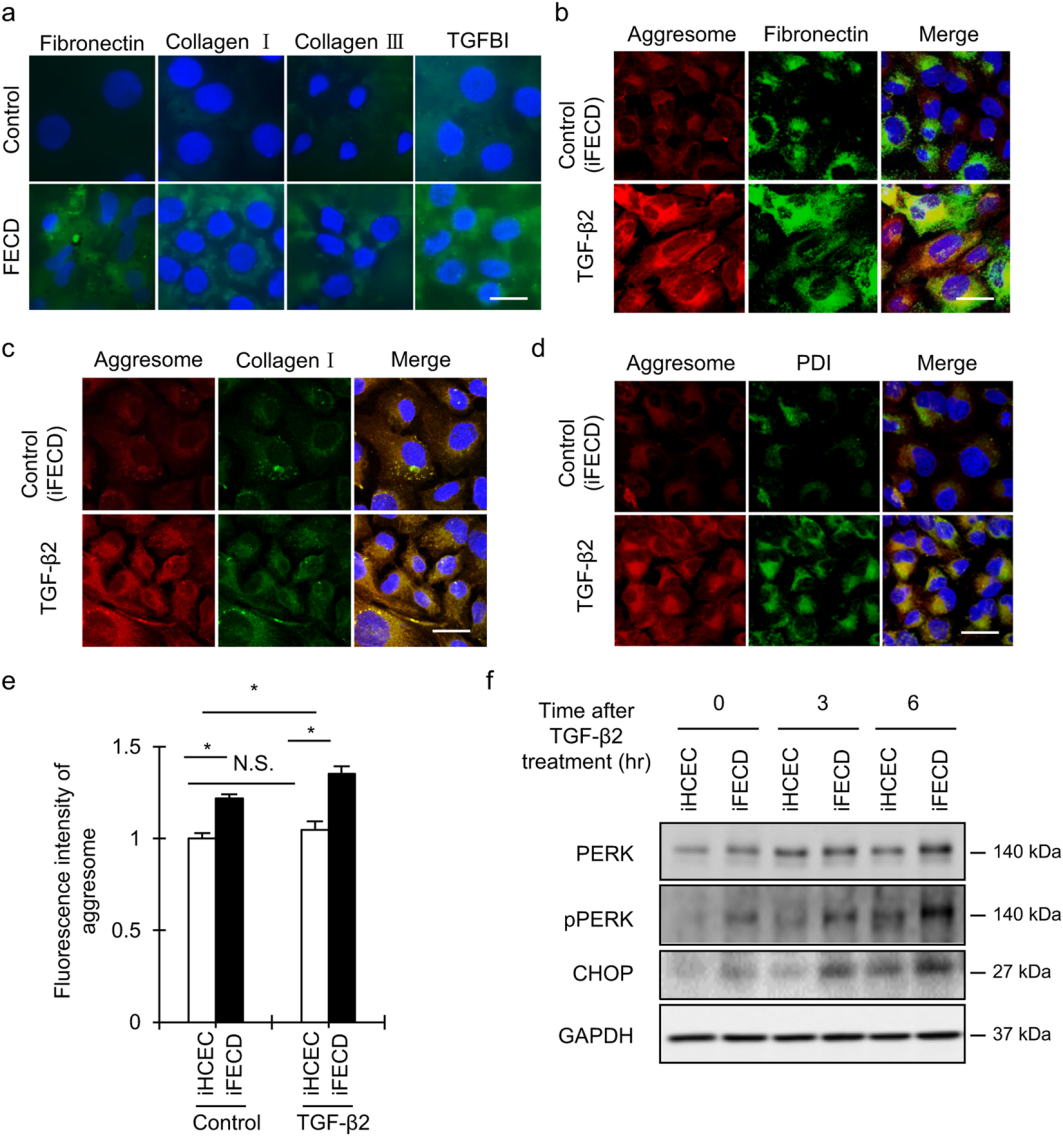
Effect of TGF-β on extracellular matrix (ECM) protein expression and unfolded protein formation. (**a**) Descemet’s membranes including CECs were obtained from 22 patients with FECD during Descemet’s membrane endothelial keratoplasty (DMEK) and were examined for the expression of ECM; fibronectin, collagen type 1, collagen type 3, and TGBI were evaluated by immunofluorescent staining. Human donor corneas from 19 non-FECD subject were used as control. Scale bar: 50 µm. (**b**,**c**) iFECD, which is a corneal endothelial cell model established from patients with FECD, were cultured and stimulated with TGF-β2 for 48 hours. The expression of fibronectin and collagen type 1 was evaluated by immunofluorescence staining. Unfolded protein was assessed by aggresome staining. Nuclei were stained with DAPI. Experiments were performed in triplicate. Scale bar: 50 µm. (**d**) Immunofluorescent staining of PDI and aggresome staining was performed to evaluate the codistribution of endoplasmic reticulum (ER) and unfolded protein. Nuclei were stained with DAPI. Experiments were performed in triplicate. Scale bar: 50 µm. (**e**) iFECD and iHCEC were cultured with or without TGF-β2 (10 ng/ml) for 48 hours. Cells were stained with aggresome and fluorescence intensity was evaluated by flow cytometry. Experiments were performed in triplicate. The statistical analysis was performed by Student’s t-test. *P < 0.01. (**f**) iFECD and iHCEC were cultured with or without TGF-β2 (10 ng/ml), and expression of PERK, phosphorylation of PERK, and expression of CHOP was evaluated by western blotting. Experiments were performed in triplicate. GAPDH was used as internal control.

Descemet’s membranes, including CECs, were obtained from patients with FECD who underwent Descemet’s membrane endothelial keratoplasty (DMEK). The membranes were analyzed for the expression of ECM components by immunofluorescent staining. Expression of fibronectin, collagen type 1, collagen type 3, and transforming growth factor beta induced (TGFBI) was evident in the FECD specimens, especially in the cytoplasm of endothelial cells neighboring the guttae, but no or very faint expression of those components was observed in control specimens (Fig. 2a). We also evaluated the effect of TGF-β on ECM components and unfolded protein formation by using iFECD, which is a corneal endothelial cell model established from patients with FECD. Fibronectin and collagen type 1 were expressed in the cytoplasm in iFECD cells at levels similar to those observed in patient specimens. Aggresome staining showed that unfolded protein forms aggregates and fibronectin and collagen type 1 were at least partially colocalized to the aggresome. TGF-β treatment increased the expression of fibronectin and collagen type 1, as well as aggresome formation (Fig. 2b,c). Co-staining for the aggresome and protein disulfide isomerase (PDI, marker of ER) demonstrated that the aggresomes were localized to the ER (Fig. 2d). Flow cytometry showed that the intensity of aggresome staining was significantly higher in iFECD than in iHCEC; the latter is a cell model established from non-FECD human donor corneas. Notably, TGF-β stimulation caused an upregulation of the aggresome only in iFECD but not in iHCEC (Fig. 2e). Consistently, TGF-β induced a higher phosphorylation of PKR-like endoplasmic reticulum kinase (PERK), which is an ER sensor responsible for ER-mediated apoptosis, and increased expression of CCAAT-enhancer-binding protein homologous protein (CHOP), which is a transcription factor that transduces ER mediated apoptotic signals to the mitochondria, in iFECD than in iHCEC (Fig. 2f).

### Activation of ER stress and the intrinsic apoptotic pathway by TGF-β

**Figure 3:**
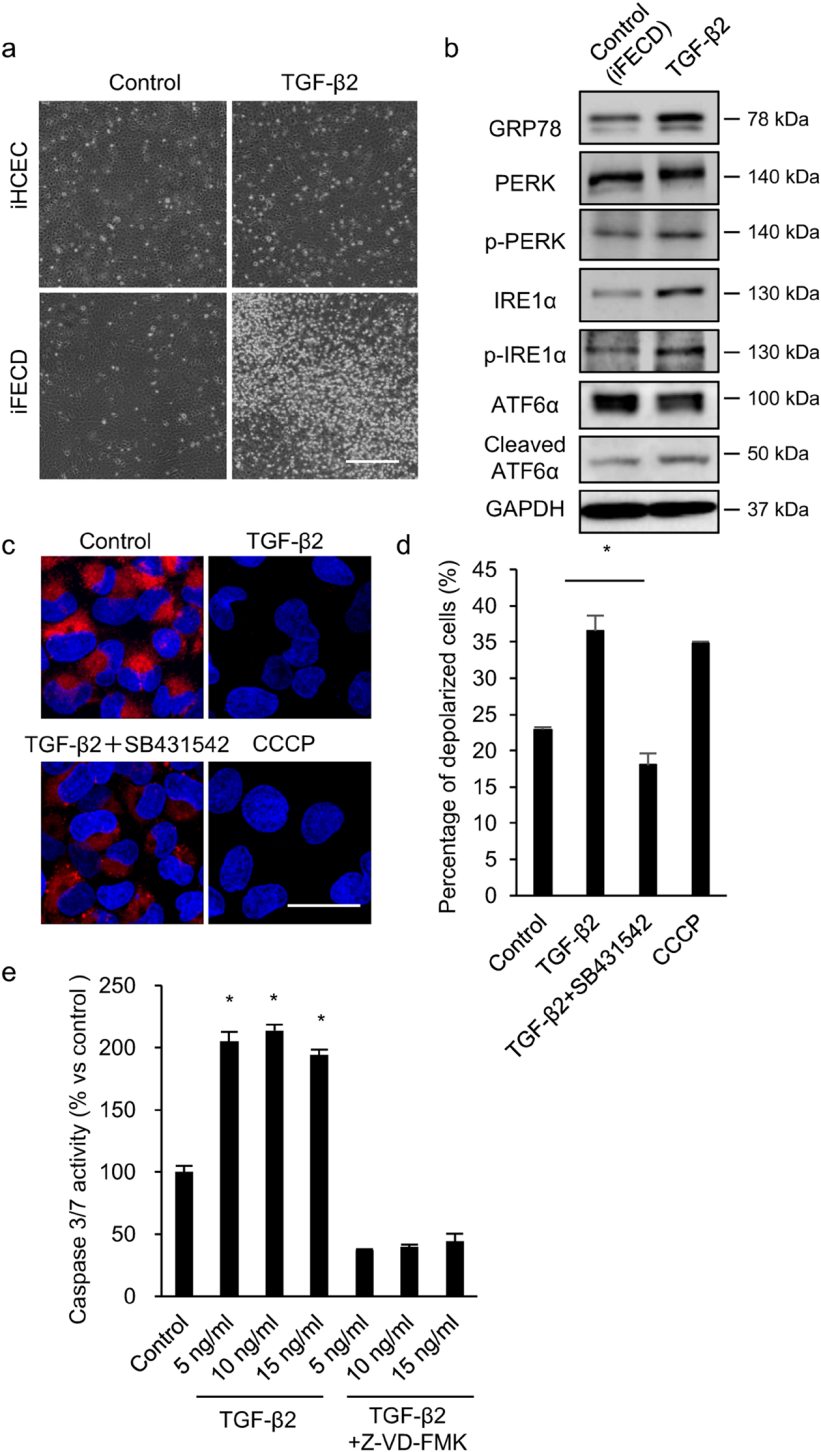
Activation of endoplasmic reticulum (ER) stress and subsequent intrinsic apoptotic pathway by TGF-β. (**a**) iFECD and iHCEC was cultured with or without TGF-β2 (10 ng/ml) for 48 hours. Representative phase contrast images are shown. Experiments were performed in triplicate. Scale bar: 200 µm. (**b**) iFECD was treated with TGF-β2 for 6 hours and expression of GRP78, PERK, IRE1α, and phosphorylation of PERK and IRE1α, and cleavage of ATF6α were evaluated by western blotting. Full-length blot of ATF6α is presented in Supplementary Fig. 1. GAPDH was used as internal control. Experiments were performed in triplicate. (**c**,**d**) iFECD was treated with TGF-β2, TGF-β2 + SB431542 for 6 hours. After JC-1 staining, mitochondrial membrane potential (MMP) depolarization was evaluated by fluorescence microscopy and flow cytometry. Nuclei were stained with DAPI for fluorescence microscopy analysis. CCCP was used as positive control to induce MMP depolarization. Experiments were performed in triplicate. Scale bar: 50 µm. The statistical analysis was performed by Student’s t-test. *P < 0.01. (**e**) iFECD was treated with TGF-β2 (5, 10, or 15 ng/ml) for 6 hours with or without caspase inhibitor, Z-VD-FMK, then caspase 3/7 activity was determined by use of the Caspase 3/7 activity assay^®^ Luminescent Assay. Experiments were performed in duplicate. The statistical analysis was performed by Dunnett’s multiple comparisons test. *P < 0.01.

Phase contrast images showed that a greater number of cells was detached from the culture plate after 48 hours of TGF-β treatment in iFECD, whereas no cell detachment was evident in iHCEC after TGF-β treatment (Fig. 3a). These observations were consistent with the finding that TGF-β induced unfolded protein and subsequent ER stress only in iFECD (Fig. 2f). Western blotting showed that TGF-β induced expression of glucose-regulated protein78 (GRP78), phosphorylation of PERK and inositol-requiring enzyme 1α (IRE1α), and cleavage of activating transcription factor 6α (ATF6α) in iFECD, indicating that TGF-β induces unfolded protein and subsequent activation of all three ER sensors (Fig. 3b). Staining with 5, 5′, 6, 6′-tetrachloro-1, 1′, 3, 3′-tetraethylbenzimidazolylcarbocyanineiodide (JC-1) showed that the mitochondrial membrane potential (MMP) was depolarized by TGF-β treatment. Co-treatment with TGF-β and SB431542 suppressed the downregulation of MMP by TGF-β, suggesting that MMP depolarization was induced by activation of TGF-β signaling. Carbonylcyanide m-chlorophenylhydrazone (CCCP) was used as positive control to depolarize the MMP (Fig. 3c). Consistent with fluorescence microscopy, flow cytometry showed that TGF-β significantly upregulated the percentages of MMP depolarized iFECD when compared to the control iFECD (36.5 ± 2.1% and 22.9 ± 0.3%, respectively) (Fig. 3d). TGF-β also induced caspase 3/7 activation, which was repressed by the pan-caspase inhibitor, Z-VD-FMK (Fig. 3e). Taken together, these data suggest that TGF-β induces ER stress and activates the intrinsic apoptotic pathway in iFECD.

### Effect of a chemical chaperone on ER stress and intrinsic apoptotic pathway

**Figure 4:**
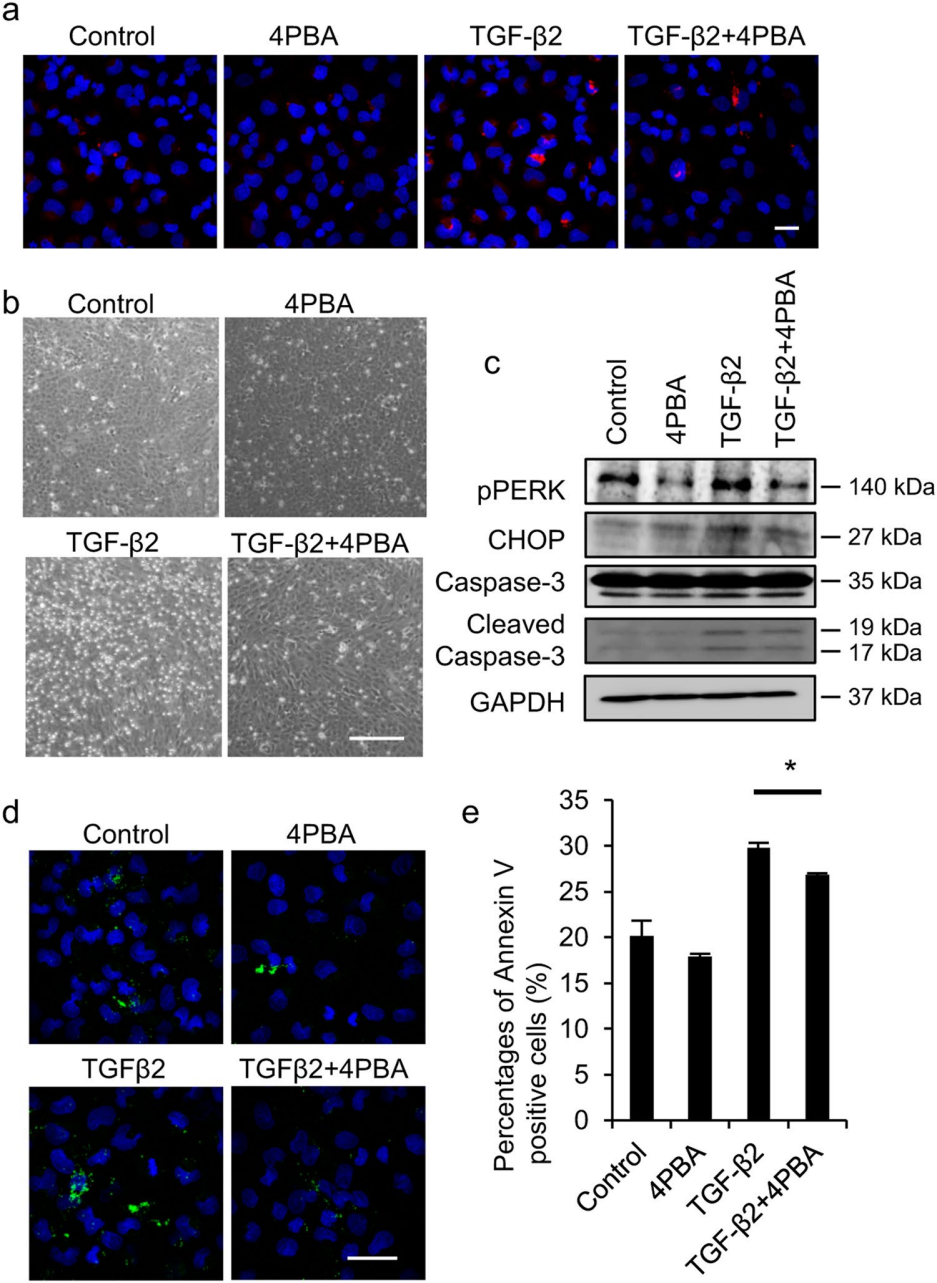
Effect of chemical chaperon on endoplasmic reticulum (ER) stress and intrinsic apoptotic pathway. (**a**) iFECD was treated with or without TGF-β2 (10 ng/ml) for 24 hours, and 4 PBA (5 mM) was added to the medium to evaluate the effect of a chemical chaperone on unfolded protein formation. Unfolded protein was evaluated by aggresome staining and nuclei were stained with DAPI. Experiments were performed in triplicate. Scale bar: 50 µm. (**b**) Representative phase contrast images are shown. TGF-β2 induces cell detachment of iFECD, but 4 PBA suppressed the cell detachment. Experiments were performed in triplicate. Scale bar: 200 µm. (**c**) Effect of chemical chaperon on ER stress and apoptosis was evaluated. TGF-β2 treated samples were recovered after coincubation with 4 PBA and phosphorylation of PERK, expression of CHOP, and cleavage of caspase 3 were examined by western blotting. Full-length blot of caspase 3 is presented in Supplementary Fig. 1. GAPDH was used as internal control. Experiments were performed in triplicate. (**d**,**e**) Apoptotic cell were evaluated by Annexin V staining and evaluated by fluorescent microscope and flow cytometry. Nuclei were stained with DAPI for fluorescence microscopy analysis. Experiments were performed in triplicate. Scale bar: 50 µm. The statistical analysis was performed by Student’s t-test. *P < 0.01.

We also evaluated the effect of a chemical chaperone on ER stress and subsequent intrinsic apoptotic pathway activation. Aggresome staining showed that the chemical chaperone 4-phenylbutyric acid (4 PBA) repressed the formation of TGF-β mediated unfolded protein (Fig. 4a). Phase contrast microscopy showed that TGF-β induced cell detachment of iFECD, which was suppressed by 4 PBA (Fig. 4b). Consistently, TGF-β mediated phosphorylation of PERK, expression of CHOP, and cleavage of caspase 3 were suppressed by 4 PBA (Fig. 4c). Annexin V staining also demonstrated the presence of apoptotic cells in TGF-β treated iFECD, but 4 PBA suppressed the occurrence of Annexin V positive cells (Fig. 4d). Flow cytometry also demonstrated that 29.8 ± 0.6% of the iFECD cells were Annexin V positive following TGF-β treatment, but 4 PBA significantly reduced the percentage of Annexin V positive cells to 26.9 ± 0.2% (Fig. 4e). These results suggest that suppression of unfolded protein formation or accumulation counteracted the UPR and subsequent intrinsic apoptotic pathway activation.

### Feasibility of inhibiting TGF-β signaling pathway as therapeutic target for the treatment of FECD

**Figure 5:**
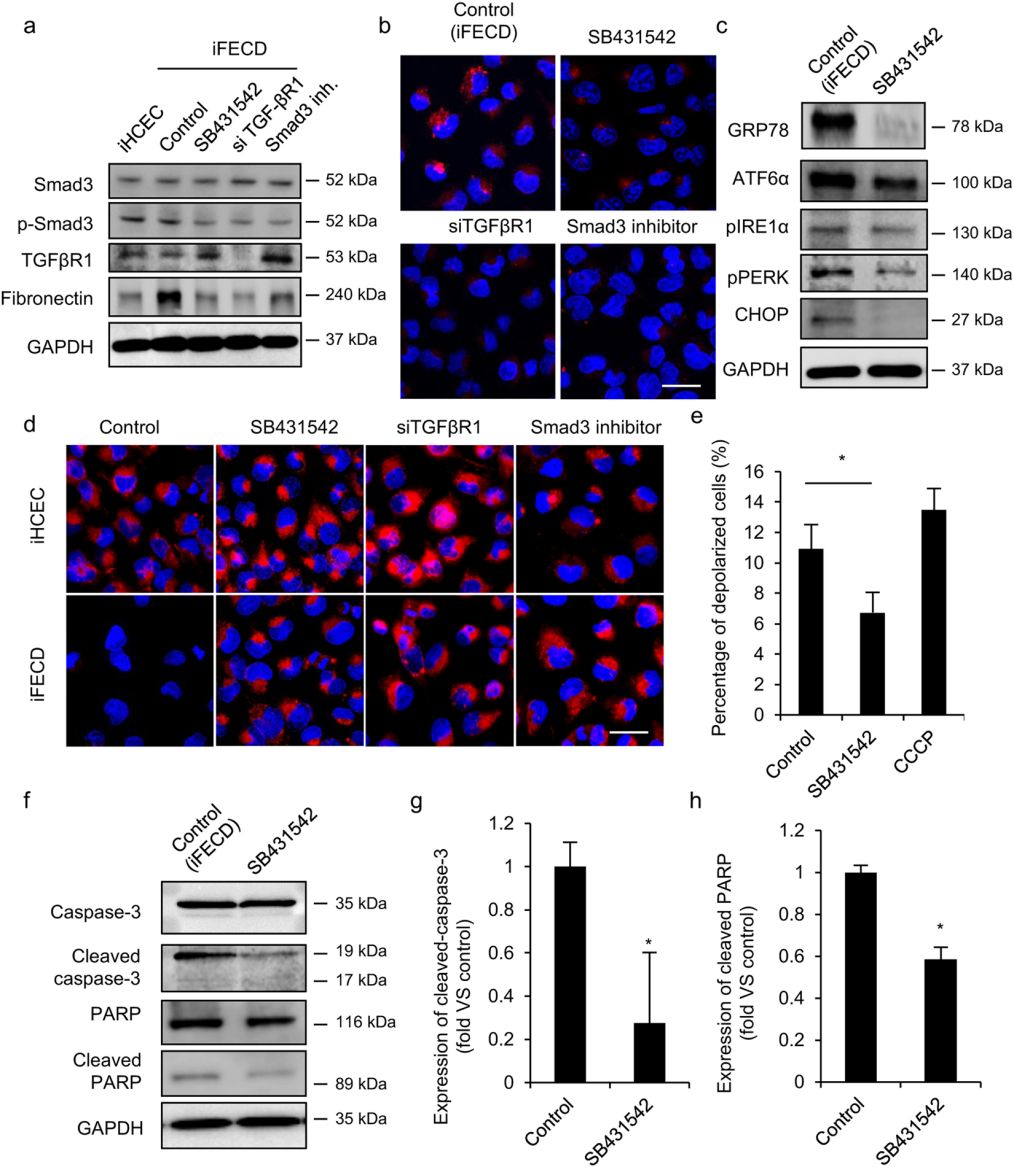
Effect of inhibiting TGF-β signaling pathway on endoplasmic reticulum (ER) stress mediated cell death. The effect of inhibiting TGF-β signaling pathway on extracellular matrix (ECM) production was evaluated. iFECD was cultured with SB431542 (TGF-βR1 inhibitor) or Smad inhibitor, or knockdown TGF-βR1 by siRNA. Phosphorylation of Smad3 was suppressed by SB431542, knockdown TGF-βR1, and Smad inhibitor. Production of fibronectin by iFECD was evaluated by western blotting. Experiments were performed in duplicate. (**b**) TGF-β signaling pathway was blocked by SB431542 (TGF-βR1 inhibitor) or Smad inhibitor, or knockdown TGF-βR1 by siRNA. The effect of TGF-β signaling pathway inhibition on unfolded protein was evaluated by aggresome staining. Experiments were performed in triplicate. Scale bar: 50 µm. (**c**) Effect of inhibition of the TGF-β signaling pathway on endoplasmic reticulum (ER) stress. Expression of GRP78, ATF6 pIRE1α, and CHOP, and phosphorylation of IRE1α and PERK was evaluated by western blotting. GAPDH was used as internal control. Experiments were performed in triplicate. (**d**,**e**) Effect of inhibiting TGF-β signaling pathway on mitochondrial membrane potential (MMP) depolarization was evaluated by fluorescent microscope after JC-1 staining and flow cytometry. Nuclei were stained with DAPI for fluorescence microscopy analysis. CCCP was used as positive control to induce MMP depolarization. Experiments were performed in duplicate. Scale bar: 50 µm. The statistical analysis was performed by Student’s t-test. *P < 0.01. (**f**–**h**) Western blotting was preformed to evaluate the effect of inhibiting TGF-β signaling pathway on cleavage caspase 3 and PARP. Full-length blots are presented in Supplementary Fig. 1. Densitometry analysis of three independent experiments was performed with ImageJ and plotted as graphs. The statistical analysis was performed with the Student’s t-test. *P < 0.01.

We evaluated the effect of inhibiting the TGF-β signaling pathway on endothelial cell survival in iFECD. The iFECD was cultured with SB431542 (TGF-βR1 inhibitor) or a Smad inhibitor, or subjected to knockdown of TGF-βR1 by siRNA. Phosphorylation of Smad3 was suppressed by SB431542, knockdown of TGF-βR1, and the Smad inhibitor. The efficacy of knockdown of TGF-βR1 by siRNA was confirmed by western blotting. Western blotting also showed that fibronectin production was higher in iFECD than in iHCEC, as observed in the corneal endothelium of patients with FECD. On the other hand, inhibition of the TGF-β signaling pathway by all three independent methods suppressed the production of fibronectin in iFECD (Fig. 5a).

Aggresome staining demonstrated that the formation of unfolded protein was suppressed by SB431542, knockdown of TGF-βR1, and the Smad inhibitor (Fig. 5b). Expression of GRP78 was suppressed by SB431542, indicating the presence of unfolded protein in iFECD and its reduction by SB431542 treatment. Consistently, three ER sensors were downregulated by SB431542, which causes repression of CHOP (Fig. 5c). JC-1 staining showed that the MMP depolarization observed in iFECD was rescued by SB431542, knockdown of TGF-βR1, and the Smad inhibitor (Fig. 5d). Flow cytometry showed that the percentage of MMP depolarization in iFECD was 10.9 ± 0.9%, but this was suppressed to 6.7 ± 0.8% by SB431542 (Fig. 5e). CCCP was used to depolarize MMP as positive control.

We also evaluated the effect of inhibition of TGF-β signaling on the apoptosis executor molecules. Western blotting showed that activation of caspase 3 and Poly (ADP-ribose) polymerase (PARP) by cleavage was counteracted by SB431542 (Fig. 5f). Densitometry analysis of western blotting also showed a significant downregulation of apoptosis executor molecules by SB431542 (Fig. 5g,h).

## Discussion

TGF-β regulates a wide range of key events in cell proliferation, cell differentiation, wound healing, and immunity. Hence, it is also involved in different forms of pathogenesis, such as fibrosis, connective tissue disorders, and cancer^21–25^. Since the first report of a profibrotic effect of TGF-β, in which subcutaneous injection of TGF-β1 induce fibrotic lesions^26^, many studies have confirmed TGF-β as an important modulator of fibrosis in many different cells and tissues^25^. TGF-β induces its profibrotic effect by promoting ECM synthesis, inhibiting collagenolysis, and enhancing the proliferation of fibroblasts during wound healing^26, 27^. TGF-β is also recognized as one of the most important regulators of epithelial-mesenchymal transition (EMT) and the endothelial to mesenchymal transition (EndMT) in several models, such as tubulointerstitial renal fibrosis, pulmonary fibrosis, and heart fibrosis^28–30^.

We reported that activation of TGF-β signaling augmented the accumulation of ECM components through activation of EMT inducer genes, such as *ZEB1* and *SNAI1*, in FECD^20^. In addition, a cell model derived from patients with FECD exhibited high responsiveness to TGF-β, as CECs established from patients with FECD produced higher amount of ECM following TGF-β treatment when compared with control CECs^20^. In the current study, we provided *in vivo* patient data showing that TGF-β isoforms; TGF-β1 and TGF-β2 and TGF-β receptors; TGF-βR1, and TGF-βR2 were highly expressed in patient corneal endothelium. To the best of our knowledge, this is the first data from patient tissue sample to show that the TGF-β signaling pathway is upregulated and may play an important role in FECD. The higher expression TGF-β receptors could also be a reasonable explanation for the strong responsiveness of FECD-CECs to TGF-β, as previously reported^20^.

Unfolded protein accumulation in the ER lumen is triggered by several causes, such as nascent protein increases, mutations, shortages of chaperones, and oxidative stress^7, 8, 10, 11^. We showed that TGF-β induced fibronectin and type 1 collagen synthesis associated with the formation of unfolded protein. Interestingly, TGF-β mediated UPR and intrinsic apoptotic-signaling activation was more evident in iFECD than in iHCEC. This finding is consistent with our previous findings, in which iFECD exhibited higher responsiveness to TGF-β^20^, and with our current data in which TGF-β receptors were expressed at higher levels in patient samples. Further studies are needed to elucidate whether TGF-β induces the UPR by recruiting other proteins, such as type 3 collagen, clusterin, TGFBI, and proteoglycans, which are known to accumulate in FECD, similar to fibronectin and type 1 collagen^19, 31, 32^. Other possible combined mechanisms worth investigating in future research include the impairment of ERAD, which ubiquitinates and degrades unfolded proteins of the ER^9^, in FECD, resulting in the formation of unfolded protein.

We also demonstrated that inhibition of the TGF-β signaling pathway suppressed aggresome accumulation and UPR, and subsequently repressed apoptotic signaling activation. The TGF-β signaling pathway has been an attractive therapeutic target in various diseases^21–25^. A possible side effect of TGF-β signaling inhibition is worsening of autoimmune disease due to the inhibition of regulatory T cells role; however, TGF-β inhibition has been generally considered to have limited adverse effects^24, 33^. Several potential therapeutic strategies have been proposed to inhibit the TGF-β signaling pathway, such as (1) neutralizing antibodies or soluble receptors to TGF-β, (2) receptor kinase inhibitors, (3) Smad3 inhibitors, (4) Smad7 agonists, and (5) histone deacetylase inhibitors^24, 33^. At present, clinical trials of anti-TGF-β antibodies and TGF-β receptor inhibitors have been performed for a numbers of cancers, such as renal cell carcinoma, melanoma, glioma, glioblastoma, hepatocellular carcinoma, pancreatic ductal carcinoma and ovarian cancers^24, 33^. For targeting fibrotic disease, neutralizing antibodies for TGF-β were used for the treatment of primary focal segmental glomerulosclerosis and diabetic kidney disease^34–36^.

**Figure 6:**
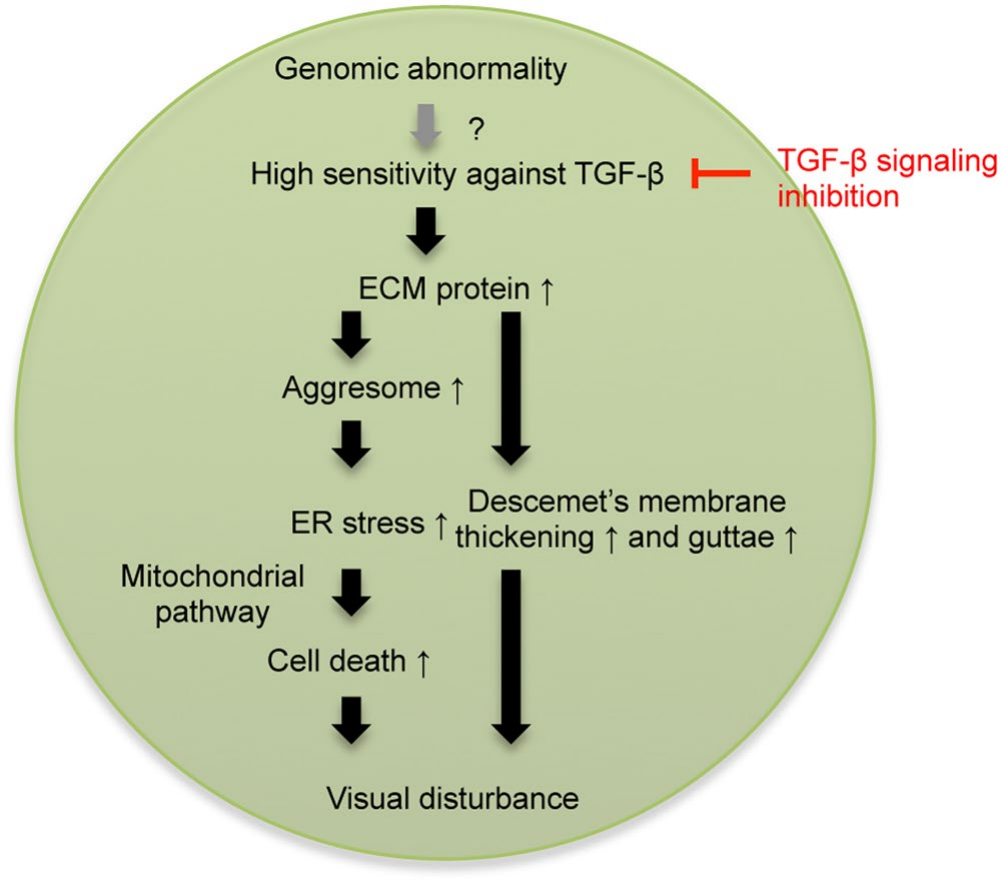
Schematic image shows possible pathophysiology of FECD. High sensitivity to TGF-β and activation of TGF-β signaling pathway induce excessive production of ECM components. ECM components form clinically phenotypic features, namely Descemet’s membrane thickening and guttae formation. At the same time, the overload of ECM proteins will form unfolded protein and trigger the UPR, and the cells will undergo apoptosis through the intrinsic pathway. We also showed that TGF-β signaling pathway might be a possible therapeutic target for treatment of FECD.

In conclusion, we provided evidence that the expression level of TGF-β isoforms and TGF-β receptors are high in the corneal endothelium of patients with FECD. We propose that TGF-β signaling mediates a chronic overload of proteins to the ER, which enhances the formation of unfolded protein and activates the intrinsic apoptotic pathway through the UPR (Fig. 6). The inhibition of TGF-β signaling may therefore represent a therapeutic target for suppressing cell loss as well as for accumulation of ECM in FECD.

## Methods

### Ethics Statement

The human tissue used in this study was handled under the guidelines based on the ethical principles of the Declaration of Helsinki. This study was performed according to a protocol approved by the ethical review committee of the Friedrich-Alexander University Erlangen-Nürnberg. Informed consent was acquired from patients with FECD, then stripped Descemet’s membranes with CECs were obtained during DMEK at University of Erlangen-Nürnberg. Normal human donor corneas were obtained from SightLife^TM^ (http://www.sightlife.org/, Seattle, WA). No tissues were procured from prisoners.

### Quantitative Real-Time PCR

Total RNA was extracted from the corneal endothelium of the FECD patient and donor corneas, and cDNA was synthesized by utilizing SuperScript^TM^ VILO^TM^ MasterMiX (Thermo Fisher Scientific Inc., Waltham, MA, USA). Quantitative real-time PCR was performed with a StepOnePlus™ Real-Time PCR System (Thermo Fisher Scientific Inc.). All samples were analyzed in duplicate with a program of 95 °C for 20 seconds and 40 cycles of 95 °C for 1 seconds and 60 °C for 20 seconds. For quantification, standard curves using serial dilutions (5–5^5^ copies) of iHCEC cDNA were run in parallel. Ratios relative to GAPDH were calculated for normalization of gene expression levels.

Gene expression levels were analyzed using TaqMan^®^ real-time PCR (Thermo Fisher Scientific Inc.). Taqman primers for *TGF*-*β1*, Hs00998133_ml; *TGF*-*β2*, Hs00234244_ml; *TGFβR1*, Hs00610320; *TGFβR2*, Hs00234253_ml; and TaqMan^®^ pre-development human GAPDH (Thermo Fisher Scientific Inc.) were used.

### Immortalization of CECs

The CECs from three patients with FECD and three donor corneas were cultured and immortalized as a cell model. Descemet’s membranes, including the CECs, were stripped from three normal human donor corneas and from corneas of three patients with FECD during DMEK. The CECs were cultured and immortalized according to modified published protocols. Briefly, Descemet’s membranes containing the HCECs were digested with 1 mg/mL collagenase A (Roche Applied Science, Penzberg, Germany) at 37 °C for 12 hours. The CECs were seeded in one well of a 48-well plate coated with laminin E8 fragments (iMatrix-511; Nippi, Incorporated, Tokyo, Japan)^37^ and were cultured by culture medium for CECs.

The culture medium was prepared according to published protocols^20, 38^. First, human bone marrow mesenchymal stem cells (BM-MSCs) were plated at a density of 1.3 × 10^4^ cells/cm^2^ and cultured for 24 hours in Dulbecco’s modified Eagle’s medium (DMEM; Life Technologies Corp.) supplemented with 10% fetal bovine serum (FBS), 100 U/mL penicillin, and 100 μg/mL streptomycin. The culture medium was then replaced with the following: OptiMEM-I (Life Technologies Corp., Carlsbad, CA) containing 8% FBS, 5 ng/mL epidermal growth factor (Sigma-Aldrich Co., St. Louis, MO), 20 μg/mL ascorbic acid (Sigma-Aldrich Co.), 200 mg/L calcium chloride, 0.08% chondroitin sulfate (Wako Pure Chemical Industries, Ltd., Osaka, Japan), 50 μg/mL gentamicin, and conditioned by culturing BM-MSCs for 24 hours. Finally, the conditioned medium with BM-MSCs was collected for use as the culture medium for CECs.

CECs from patients with FECD and from donor corneas were cultured in the culture medium for CECs for 14–21 days and then immortalized using both SV40 and hTERT, according to published protocols. Immortalized CECs from patients with FECD and donor corneas were referred to as iFECD and iHCEC, respectively. The immortalized cells were cultured in DMEM containing 10% FBS and 1% penicillin and streptomycin (Life Technologies Corp.). Once the cells were 80% confluent, the cells were trypsinized with 0.05% Trypsin-EDTA and passaged.

The iFECD or iHCEC were cultured until confluent and further cultured with fresh DMEM supplemented with TGF-β2 for 24 hours for some experiments. The effect of a chemical chaperone was evaluated by culturing with DMEM supplemented with 4 PBA (5 mM, Merck Millipore, Billerica, Massachusetts, USA). To evaluate the effect of TGF-β signaling inhibition, the cells were incubated with 10 μM SB431542 (Merck Millipore) or 10 μM Smad 3 inhibitor (Santa Cruz Biotechnology, Santa Cruz, CA, USA). The siRNA for TGFβ R1 (Thermo Fisher Scientific Inc.) was also used to suppress TGF-β signaling. Briefly, cells were seeded into a 24-well plates and cultured until they reached 50–70% confluence. The cells were then incubated with RNAi duplex (TGFβR1) and Lipofectamine™RNAiMAX (Thermo Fisher Scientific).

### Immunofluorescence staining

Descemet’s membranes, including the CECs, derived from 22 patients with FECD and 19 donor corneas, and cultured iHCEC and iFECD underwent immunofluorescence and aggresome staining. Samples were fixed for 20 minutes with 4% paraformaldehyde, permeabilized with 0.5% Triton^**®**^ X-100 (Nacalai Tesque, Kyoto, Japan), and then incubated with 1% bovine serum albumin (BSA) to block nonspecific binding. Samples were incubated with primary antibodies against fibronectin (1:200; Merck Millipore), collagen I (1:200; Merck Millipore), collagen III (1:100; abcam, Cambridge, UK), TGFBI (1:500; abcam), and PDI (1:200; Cell Signaling Technology, Danvers, Massachusetts, USA) overnight at 4 °C. Alexa Fluor^®^ 488-conjugated goat anti-mouse (Life Technologies Corp.) antibodies were used as secondary antibodies at a 1:1000 dilution at room temperature for 1 hours. Nuclei were stained with DAPI (Vector Laboratories, Burlingame, CA, USA). The slides were examined with a fluorescence microscope (DM 2500; Leica Microsystems, Wetzlar, Germany).

### Aggresome staining

Samples were stained with aggresome and aggregation of unfolded protein was evaluated by fluorescence microscope and flow cytometry according to the manufacturer’s protocol. For fluorescence microscope analysis, samples were incubated with aggresome reagent (1:2000; Enzo Life Science Inc., Farmingdale, NY, USA) for 60 minutes and slides were examined with a fluorescence microscope (DM 2500). For flow cytometry, cells were washed twice with phosphate buffered saline (PBS) and incubated with Accumax^TM^ (Innovative Cell Technologies, San Diego, CA, USA) for 10 minutes at 37 °C. Cells were recovered in fluorescence activated cell sorting (FACS) buffer composed of DMEM without Phenol Red (Nacalai Tesque) + 2%FBS, passed through a BD Falcon™ 70 μm cell strainer (BD Biosciences, Franklin Lakes, New Jersey, USA), and resuspended in FACS buffer. The cells were then incubated with aggresome reagent (1:2000) for 60 minutes at room, temperature, and washed three times with PBS. The cells were resuspended in FACS buffer and analyzed by flow cytometry using CellQuest Pro software (BD Biosciences).

### Immunoblotting

The cells were washed with ice-cold PBS and lysed with ice-cold radio-immunopreci-pitation assay (RIPA) buffer containing phosphatase inhibitor cocktail 2 (Sigma-Aldrich Co.) and protease inhibitor cocktail (Roche Applied Science). Following centrifugation, the supernatant containing the total proteins was fractionated by sodium dodecyl sulfate (SDS)-poly-acrylamide gel electrophoresis (PAGE). The separated proteins were transferred to polyvinylidene difluoride (PVDF) membranes, blocked with 3% non-fat dry milk, and incubated overnight at 4 °C with the following primary antibodies: PERK (1:1000; Cell Signaling Technology), phosphorylated PERK (1:1000; Cell Signaling Technology), PERK (1:1000; Cell Signaling Technology), IRE1α (1:1000; Cell Signaling Technology, phosphorylated IRE1α (1:1000; Novus Biologicals, Colorado, USA), ATF6α (1:1000; Santa Cruz Biotechnology), CHOP (1:1000; Cell Signaling Technology), GRP78 (1:1000; Santa Cruz Biotechnology), Smad3 (1:1000; Cell Signaling Technology), phosphorylated Smad3 (1:1000; Cell Signaling Technology), TGFβR1 (1:1000; Santa Cruz Biotechnology), fibronectin(1:1000; BD Biosciences), caspase-3 (1:1000; Cell Signaling Technology), PARP (1:1000; Cell Signaling Technology), and GAPDH (1:2000; Medical & Biological Laboratories Co., Ltd., Aichi, Japan). The blots were probed with horseradish peroxidase-conjugated secondary antibodies (1:5000; GE Healthcare, Piscataway, NJ, USA) and developed with luminal for enhanced chemiluminescence using the ECL Advanced Western Blotting Detection Kit (Nacalai Tesque).

### Mitochondrial membrane potential

The iFECD and iHCEC were stained with 5, 5′, 6, 6′-tetrachloro-1, 1′, 3, 3′-tetraethylbenzimidazolylcarbocyanine iodide (JC-1) and the mitochondrial membrane potential was evaluated by fluorescence microscopy and flow cytometry according to the manufacturer’s protocol. For fluorescence microscopy analysis, the cells were incubated with MitoScreen (10 μM; Merck Millipore) for 15 minutes, fixed with 4% formaldehyde for 10 minutes, and the slides were examined with a fluorescence microscope (DM 2500; Leica Microsystems). For flow cytometry, cells were recovered in FACS buffer and incubated with MitoScreen (10 μM; Merck Millipore) for 15 minutes at 4 °C, washed three times with PBS, resuspended in FACS buffer, and analyzed by flow cytometry using CellQuest Pro software (BD Biosciences).

### Caspase 3/7 activity assay

iFECD were seeded at a density of 5.0 × 10^3^ cells/cm^2^ per well on a 96-well plate for 24 hours, and were stimulated with TGF-β2 by culturing DMEM supplemented with TGF-β2 (5, 10, and 15 ng/ml) for 24 hours. As a control, iFECD were treated with a pan-caspase inhibitor, Z-VD-FMK (10 μM, Wako Pure Chemical Industries, Ltd.). The activity of caspase 3/7 was determined by the Caspase 3/7 activity assay^®^ Luminescent Assay performed in accordance with the manufacturer’s protocol using a Veritas™ Microplate Luminometer (Promega). Four samples were prepared for each group.

### Annexin V assessment

Cell apoptosis was evaluated with Annexin V (Medical & Biological Laboratories Co., Ltd.) staining. For fluorescence microscopy analysis, the cells were incubated with DMEM supplemented with Annexin V for 30 minutes, followed by fixation with 4% paraformaldehyde for 10 minutes. The slides were examined with a fluorescence microscope (DM 2500; Leica Microsystems). For flow cytometry, cells were incubated with DMEM supplemented with Annexin V for 15 minutes, and harvested by digestion with Accumax^TM^ (Innovative Cell Technologies). Recovered cells were then analyzed by flow cytometry using CellQuest Pro software (BD Biosciences).

### Statistical Analysis

Statistical analysis was performed with the Excel software program (Microsoft Corporation, Redmond, Washington). The statistical significance (*P*-value) in mean values of the two-sample comparison was determined with the Student’s t-test or Mann–Whitney U test. The statistical significance for the comparison of multiple sample sets was determined with Dunnett’s multiple-comparisons test. A P-value of <0.05 was considered statistically significant. Values shown represent the mean ± standard error of the mean (SEM).

## Electronic supplementary material

Supplemental Figure 1
