# Supplementary material for: Activation of TGF-β signaling induces cell death via the unfolded protein response in Fuchs endothelial corneal dystrophy
Source: Sci Rep. 2017 Jul 28;7:6801. doi: 10.1038/s41598-017-06924-3 (PMC5533742; doi:10.1038/s41598-017-06924-3)
Supplement: Supplementary file 1 — Supplemental Figure 1 [file 41598_2017_6924_MOESM1_ESM.pdf]

Supplementary information

Activation of TGF-β signaling induces cell death via the unfolded protein response in Fuchs endothelial corneal dystrophy

N Naoki Okumura[1], Keisuke Hashimoto[1], Miu Kitahara[1], Hirokazu Okuda[1], Emi Ueda[1], Kyoko Watanabe[1], Makiko Nakahara[1], Takahiko Sato[2], Shigeru Kinoshita[3], Theofilos Tourtas[4], Ursula Schlötzer-Schrehardt[4], Friedrich Kruse[4], and Noriko Koizumi[1]

[1]Department of Biomedical Engineering, Faculty of Life and Medical Sciences, Doshisha University, Kyotanabe, Japan
[2]Department of Ophthalmology, Kyoto Prefectural University of Medicine, Kyoto, Japan;
[3]Department of Frontier Medical Science and Technology for Ophthalmology, Kyoto Prefectural University of Medicine, Kyoto, Japan and 4Department of Ophthalmology, University of Erlangen-Nürnberg, Erlangen, Germany

Corresponding author
Noriko Koizumi, M.D., Ph.D., Department of Biomedical Engineering, Faculty of Life and Medical Sciences, Doshisha University, Kyotanabe 610-0321, Japan.
E-mail: nkoizumi@mail.doshisha.ac.jp
Tel and Fax: +81-774-65-6125

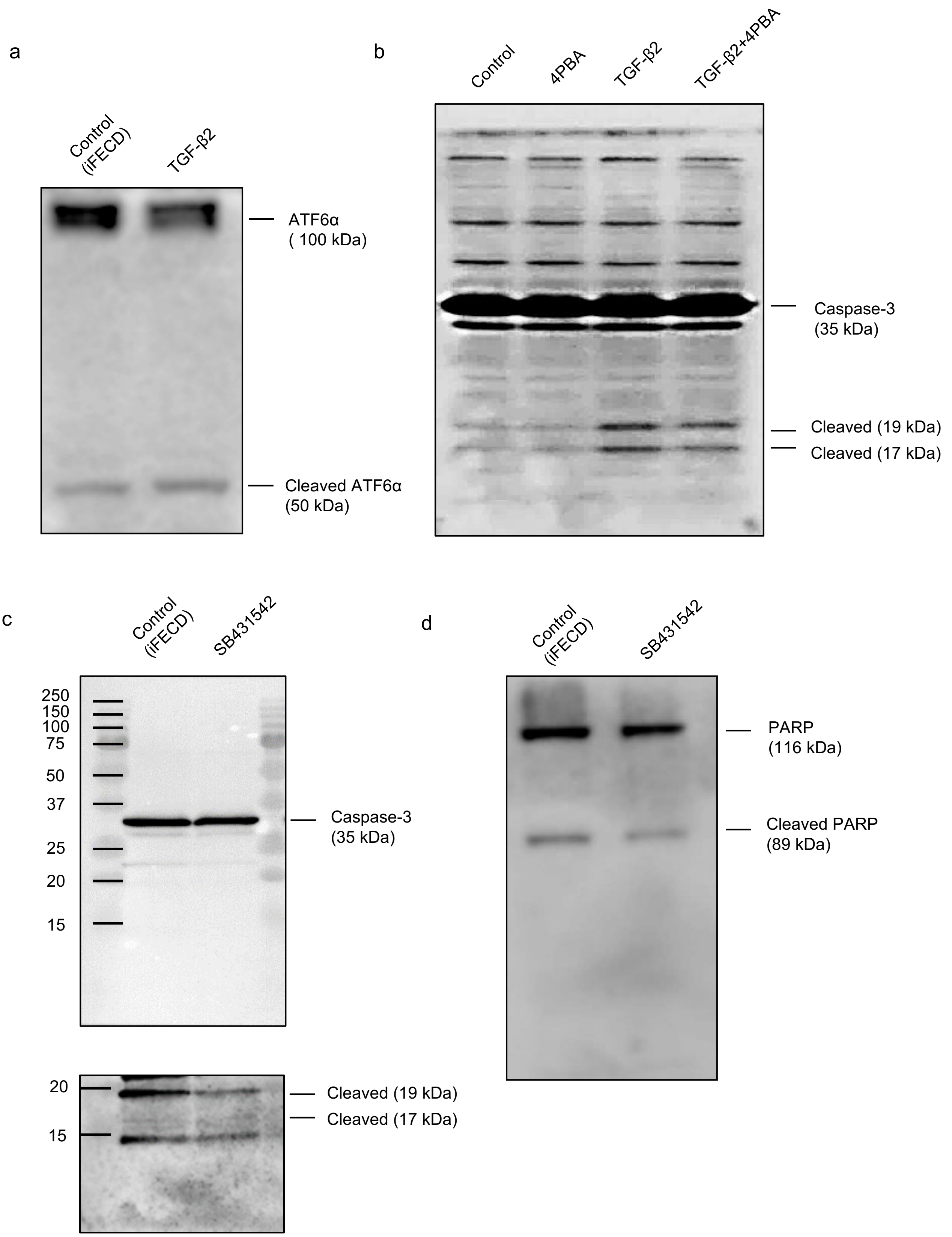

Supplemental Figure 1. Full western blots of proteins, including their cleaved forms. Samples are those shown in (a) Fig 3b, (b) Fig 4c, and (c and d) Fig 5F.
